# Cumulative Risk Assessment and Environmental Equity in Air Permitting: Interpretation, Methods, Community Participation and Implementation of a Unique Statute

**DOI:** 10.3390/ijerph8114140

**Published:** 2011-11-04

**Authors:** Kristie M. Ellickson, Sarah M. Sevcik, Shelley Burman, Steven Pak, Frank Kohlasch, Gregory C. Pratt

**Affiliations:** Minnesota Pollution Control Agency, 520 Lafayette Road, St. Paul, MN 55155, USA; E-Mails: sarah.sevcik@state.mn.us (S.M.S.); shelley.burman@state.mn.us (S.S.); steven.pak@state.mn.us (S.P.); frank.kohlasch@state.mn.us (F.K.); gregory.pratt@state.mn.us (G.C.P.)

**Keywords:** cumulative risk assessment, extant data, multiple stressors, environmental equity, air permit, hazard indicators, exposure indicators, health indicators

## Abstract

In 2008, the statute authorizing the Minnesota Pollution Control Agency (MPCA) to issue air permits was amended to include a unique requirement to analyze and consider “*cumulative levels and effects of past and current environmental pollution from all sources on the environment and residents of the geographic area within which the facility’s emissions are likely to be deposited.*” Data describing the Statute Area suggest it is challenged by environmental and socioeconomic concerns, *i.e.*, concerns which are often described by the phrase ‘environmental equity’. With input from diverse stakeholders, the MPCA developed a methodology for implementing a cumulative levels and effects analysis when issuing air permits in the designated geographic area. A Process Document was created defining explicit steps a project proposer must complete in the analysis. An accompanying Reference Document compiles all available environmental health data relevant to the Statute Area that could be identified. The final cumulative levels and effects methodology is organized by health endpoint and identifies hazard, exposure and health indices that require further evaluation. The resulting assessment is summarized and presented to decision makers for consideration in the regulatory permitting process. We present a description of the methodology followed by a case study summary of the first air permit processed through the “cumulative levels and effects analysis”.

## 1. Introduction

Cumulative risk assessment is growing and evolving, driven by scientific findings, public concern, expanded data availability, and practical experience. Communities are acutely aware that exposures are not limited to one pollutant, from one source, through one pathway at a time. Environmental health-related agencies are under pressure to be responsive to public comments, concerns, and requests based on this growing awareness. At the same time scientific investigations of cumulative risks have become increasingly complex; progressing from investigations of aggregate exposures (phthalates); exposures to multiple yet similar chemicals (organophosphate pesticides) or more complex substances (e.g., particulate matter); to, more recently, the interaction between chemical exposures and non-chemical stressors.

The word “cumulative” is used in numerous ways. In the context of human health, the characterization of “cumulative risk assessment” ranges from the incorporation of multiple sources to multiple environmental media exposures to the inclusion of existing health outcomes. Cumulative risk and related terms are defined in the EPA Framework for Cumulative Risk Assessment [1] as “the combined risks from aggregate exposures to multiple agents or stressors.” The term “stressors” in this definition is a physical, chemical, biological, or other effect that can cause an adverse response in a human or other organism or ecosystem (e.g., socioeconomic status, existing exposures, existing health outcomes, *etc.*). The word “agents” describes chemical, biological or physical exposure entities (e.g., radon, nitrogen dioxide, a virus, *etc.*). The MPCA used the EPA’s definition described above as the appropriate starting point for development of a cumulative risk assessment method due to the broad language in the statute.

In the state of Minnesota, cumulative impact analyses have been required in environmental review since 1973 under the Minnesota Environmental Policy Act [2]. This requirement was reinforced by a 2006 Minnesota State Supreme Court decision commonly referred to as the “CARD decision” (Citizens Advocating Responsible Development *vs.* Kandiyohi Board of Commissioners, [3]). This decision implies a requirement of a multi-source assessment and pursuant to this decision MPCA developed guidance [4] to implement multi-source, cumulative human health risk assessments of exposures to air pollution.

The MPCA may also request a discretionary cumulative air emission risk analysis outside of the environmental review process based on concerns about the location of a facility, pollutants emitted, public interest, *etc.* Early assessments addressed multiple pollutants, multiple sources and included some quantification of existing measured ambient air concentrations but were typically limited to the inhalation pathway. A conceptual model for this type of cumulative risk assessment is a rural setting with relatively few, isolated point sources, some regional air concentrations and a small homogeneous population. This simple model does not address the complexities now confronting regulators. For an urban setting, a more representative model might include multiple small (often unpermitted) sources (including mobile, non-point and uncharacterized sources); some regional air concentrations; and existing conditions resulting from current or historic activities (that may be imprecisely characterized and difficult to model or quantify). Further, vulnerable communities or populations which may be challenged by social, environmental, demographic and economic factors need to be addressed in a more complex urban model. Such communities typically have high population densities that are racially and socio-economically heterogeneous, and may have reduced capacity to tolerate pollutant exposures. This manuscript describes a unique Minnesota statute that calls for a comprehensive consideration of cumulative risk and community vulnerability in a central Minneapolis community.

The statute resulted from a proposal to build and operate a facility for generating electricity from the combustion of biomass at a historic site in a disadvantaged neighborhood in South Minneapolis. During the air permit application review process, public opposition to the proposal mounted, and a petition was circulated requesting additional environmental review. The permit application was eventually withdrawn and the proposed project did not go forward.

Reaction to the proposed biomass facility galvanized existing environmental concerns in the community and led to the passage of legislation in 2008 that amended the statute authorizing MPCA to issue permits [5]. After several revisions during Minnesota Legislative Session 85 [6], the amended statute calls for an extensive cumulative risk assessment from both current and past exposures, with the area described by the statute identified by the five clauses:

The agency may not issue a permit to a facility without analyzing and considering the cumulative levels and effects of past and current environmental pollution from all sources on the environment and residents of the geographic area within which the facility’s emissions are likely to be deposited, provided that the facility is located in a community in a city of the first class in Hennepin County that meets all of the following conditions:

1. *is within a half mile of a site designated by the federal government as an EPA superfund site due to residential* arsenic *contamination;*
2. a majority of the population are low-income persons of color and American Indians;
3. a disproportionate percent of the children have childhood lead poisoning, asthma, or other environmentally related health problems;
4. is located in a city that has experienced numerous air quality alert days of dangerous air quality for sensitive populations between February 2007 and February 2008; and
5. *is located near the junctions of several heavily trafficked state and county highways and two one-way streets which carry both truck and auto traffic. (Minn. Stat. § 116.07 subd. 4a)* [5]

The type and methodology of the analyses are informed by the statute language *“analyzing and considering the cumulative levels and effects of past and current environmental pollution from all sources on the environment and residents of the geographic area within which the facility’s emissions are likely to be deposited.”* The process development and decision making surrounding this language, referred to by the word “*considering,*” is described in the Methodology section of this manuscript.

This statute is unique in its requirement to consider cumulative risk and environmental equity in the context of air permitting. The statute describes one small geographic location, albeit a community with disproportionate exposure to environmental stressors including air pollutants. Some census tracts within the Statute Area are low income, based on the percent of the population with incomes less than two times the national poverty level compared to statewide averages. Some of the census tracts in the Statute Area were considered to have high minority populations where the percent non-white population was above the statewide average. These metrics are often used to prioritize areas of heightened environmental equity concern.

Following the passage of this amendment the MPCA began development of an analytical process that fit within the framework and general structure of Minnesota air permitting requirements. The name of the process, Cumulative Levels and Effects (CL&E) analysis, was taken directly from the language of the statute. To aid project proposers and MPCA staff in the first part of the process, the agency chose to use existing tools and methodologies, which focused on the individual project’s potential impacts on ambient air quality and human health risks. Within this manuscript, the term “risk” generally refers to estimates of potential cancer and non-cancer health effects. While the statute did not specifically identify a risk assessment process, the MPCA used existing tools and the assessment of risk to scope further cumulative analysis. If risks or air concentrations exceeded the screening guidelines established by MPCA, then project proposer and MPCA identified the health endpoints and corresponding environmental health data to provide information for a discussion of how a project’s potential impacts may intersect with existing environmental health conditions, especially health disparities due to other sources of environmental pollution. This additional analysis allowed the MPCA to move beyond a standard assessment of risk and meet the statute’s requirement to consider the cumulative levels and effects of current and past pollution on the community. The work and the lessons learned from the initial implementation provide an example that may be useful for other jurisdictions to incorporate into the permitting process or to scope cumulative risk assessments.

## 2. Methodology

### 2.1. Interpreting and Defining the Area Described by the Statute

**Figure 1:**
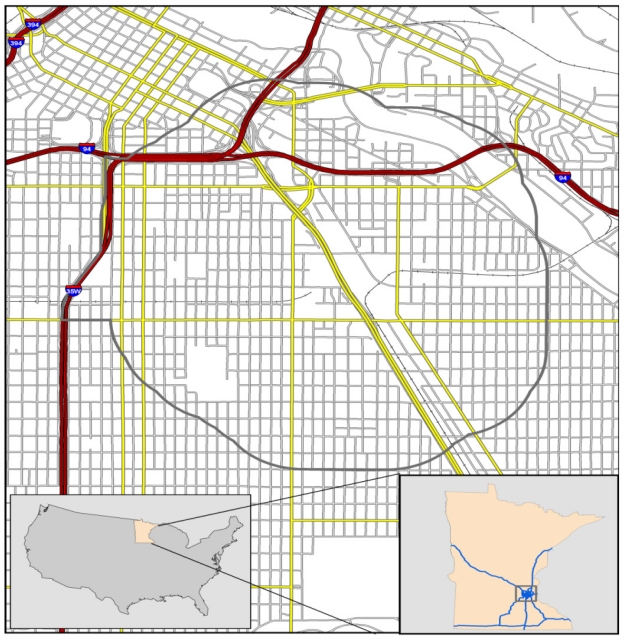
The grey outline in the figure defines an area described by Minn. Stat. § 116.07 Subd4a [5] and includes the South Minneapolis Phillips Communities and a ½ mile buffer around the Residential Soil Exposure Superfund Site.

The area described by the statute lies in the City of Minneapolis (the only *city of the first class* in Hennepin County). Additional language further limits the geographic area, notably the phrase: *is within a half mile of a site designated by the federal government as an EPA superfund site due to residential arsenic contamination* (direct statute quotes have been italicized*).* Figure 1 is a map of the Statute Area as interpreted by MPCA. Although this area is defined in statute, it is not the only area in Minnesota with disproportionately high pollutant concentrations nor is it the only area with potential environmental equity concerns. However, it is the only area that meets all the criteria specified in the statute.

### 2.2. Determining the “Geographic Area Within Which the Facility’s Emissions Are Likely to be Deposited”

Multi-pathway risk screening is conducted in regulatory risk assessments to varying degrees of refinement. For example, for large facilities or when persistent, bioaccumulative and toxic (PBTs) pollutants are emitted, pollutant deposition and multipathway risks may be specifically modeled (in addition to the standard modeling of air concentrations). Deposition modeling requires more data inputs and entails greater uncertainty than dispersion modeling of air concentrations. For these reasons, modeled air concentrations were selected as a proxy for “*emissions likely to be deposited*” rather than deposition. This area, where “*emissions are likely to be deposited*,” is called the “Study Area” and its determination is described below. The air concentration proxy is used only to scope the “Study Area” and extent of the evaluation, and not for evaluation of potential environmental effects due to pollutant deposition.

Tools developed for the MPCA regulatory risk assessment program for human health, or air emissions risk analysis (AERA), are recommended for completion of a CL&E analysis. These simple screening tools include methods whose results are summed risk values with spatial coordinates [7]. In addition to the air toxics modeling, regulatory criteria pollutant modeling is conducted following MPCA and EPA guidance. The results of the risk modeling and the criteria pollutant modeling are compared to screening levels taken from existing regulatory tools. A risk driver level (10% of the facility specific risk guideline) is used for the air toxic modeling and the Significant Impact Level (SIL) is used for criteria pollutant modeling. If any modeled concentration or risk exceeds a screening level in the ambient air, then further CL&E analysis must be completed. The Study Area is designated as a radius of the maximum distance from the facility at which a screening level is exceeded. If no modeling result exceeds a screening level, then the modeling results are submitted to the MPCA and the CL&E analysis is complete without further discussion of cumulative impacts.

### 2.3. Scoping the “Effects of Past and Current Environmental Pollution…on the Environment and Residents of the Geographic Area…”

The screening factors identified above (criteria pollutant SILs and 10% of facility risk guidelines) are also used to identify human health endpoints for inclusion in a CL&E analysis. The human health endpoint associated with a specific pollutant is determined by the toxicity values used in MPCA regulatory risk assessments. Environmental health data in the Reference Document associated with the human health endpoints (e.g., respiratory, cardiovascular, reproductive, *etc.*) for pollutants whose concentrations are above screening levels are included for further study in the CL&E analysis. Each potential health endpoint is linked to the appropriate types of environmental health data in Table 1 (taken from the Process Document). Inclusion of human health endpoints for further study in the CL&E analysis does not imply that a health impact will occur.

### 2.4. Reporting and Context for “Effects of Past and Current Environmental Pollution…on the Environment and Residents of the Geographic Area…”

Environmental health data associated with facility specific pollutant health endpoints are used to describe the existing condition or vulnerabilities in the Study Area. These environmental health data were compiled and described within a “Reference Document” [8] to provide context and to facilitate analysis and the writing of CL&E reports. These data were categorized in the same manner as the CDC Environmental Health Tracking Program (hazard indices, exposure indices and health indices) to improve readability of the Reference Document. Hazard indicators are pollutant concentrations or other surrogates for pollutant exposures. Exposure indicators are primarily biomarker data. Health indicators are comprised of statistics describing population health status. The environmental health data available for inclusion in CL&E analyses and reports are summarized in Table 1. In addition to the data types in Table 1, socioeconomic data (percent below two times statewide average for percent below poverty, percent above two times statewide average for percent non-white population, *etc.*) were collected from the US Census. Finally, more generalized data including tobacco use and percent of the population uninsured averages were collected from the Hennepin County Survey of the Health of All the Population and the Environment [9]. The data reported and described in the Reference Document are continually being updated, but are not the only data a proposer should consider. Project proposers are expected to include any additional and available data together with those data sets in the Reference Document.

### 2.5. Hazard Indicators

Traffic count data for each roadway segment in Minnesota are developed by the Minnesota Department of Transportation [10]. These data were processed using a geographic information system program (GIS program) to derive vehicle miles travelled (VMT). Traffic density was calculated from VMT by dividing the total VMT in each census tract by the tract area (miles^2^). These data provide a metric for traffic related pollutant exposures. Traffic density is used as a surrogate for actual roadway measurements to account for potential air pollution exposures from traffic to nearby residents.

The MPCA operates a statewide ambient air monitoring network with measurements of criteria pollutants, carbonyls, volatile organic compounds (VOCs), selected metals and some special project pollutants. This network is set up to show compliance with standards, to support the air quality index, to capture regional sources, community air concentration levels and in some cases potential point source impacts. Within the area described by the statute there is one ambient monitoring site where particulate matter less than 2.5 microns (PM_2.5_), metals (total and speciated), VOCs and carbonyls are measured. Maximum hourly and annual average air concentrations of carbonyls, VOCs and metals for the years from 2002–2008 were divided by toxicity benchmark concentrations to derive inhalation cancer risks, acute hazard indices and chronic hazard indices. The highest nitrogen dioxide concentration measured in the statewide network was included as an acute respiratory indicator, since NO_2_ is not specifically measured in the Statute Area.

Modeled air toxics risk results were also included as hazard indicators. The MPCA developed a risks screening tool, MNRiskS for Minnesota Risk Screening tool [11], with the support of Lakes Environmental™. In MNRiskS all inventoried pollutant emissions were modeled using an air dispersion and deposition model to generate air, water and soil concentrations of over 200 pollutants. These results were used to estimate human health risks following the Human Health Risk Assessment Protocol [12] methodology. The estimated multi-pathway cancer and non-cancer risks are spatially refined (from statewide to community level) and can be displayed geographically by pollutant or pollutant group, source or source group, or by pathway in a variety of flexible formats. MNRiskS estimates of risk were averaged by census tract as a hazardous air pollutant indicator. The National Air Toxics Assessment [13] results were also used as a separate hazardous air pollution indicator to reflect uncertainty in air toxics risk models.

The public drinking water supply in the Statute Area is operated by the City of Minneapolis, which is required to prepare an annual drinking water quality report. These data were included as descriptors of potential pollutant exposure through the drinking water pathway [14]. The MPCA, the Minnesota Department of Natural Resources and the Minnesota Department of Health maintain a fish contaminant database including fish tissue concentrations of mercury and other PBTs at numerous locations in the state. These data are used to estimate risk using assumed exposure scenarios and are also used to develop the safe eating guidelines published by the Minnesota Department of Health [15]. These data were included as a hazard indicator for the fish ingestion pathway by stating if the Study Area includes water bodies with fish advisories and estimating risks using assumed consumption rates and fish tissue concentrations.

Finally, the guidance documents outline a further analysis to identify other potential sources of pollutants located within the Statue area that are not captured by the preceding data sources. The MPCA website tool, *“What’s In My Neighborhood?”* may be used for this task [16]. Nearby sites of environmental interest may include other sources of air emissions, water permit sources, soil remediation sites, hazardous waste generators, *etc.* Some of these sources may be screened out of the analysis through examination of other regulatory controls (e.g., hazardous waste generator sites) and those sites with limited potential for human exposures. A proposed project’s impacts must be considered in the context of these other sites in the Study Area.

### 2.6. Exposure Indicators

The Minnesota Deparment of Health collects and reports blood lead concentrations in children and adults [17]. The most appropriate data for inclusion in the CL&E analysis are total counts and percentages of children under the age of 6 whose blood lead levels are above the CDC action level of 10 μg·dL^−1^. These data are available by zipcode and for comparison purposes these same data are included for the City of Minneapolis, the City of St. Paul and statewide. A qualifier must be included for the blood lead indicator since there have been observed effects below the 10 μg·dL^−1^ level.

In 2007, the Minnesota legislature directed the MDH to conduct a biomonitoring study of the area surrounding the South Minneapolis Residential Soil Exposure Site. Urine arsenic was measured during two consecutive first morning voids in 65 children. Urine concentrations higher than 15 μg·g^−1^ (creatinine corrected) were speciated to further elucidate potential sources of arsenic exposure. These data are included in the guidance document, but the data must be used with the appropriate caveats [18].

### 2.7. Health Indicators

The MDH collects and reports zip code level data on hospitalizations and emergency department visits due to asthma complications or episodes [19]. While asthma incidence tends to be quite stable statewide and nationwide, asthma related emergency room visits and hospitalizations may be disproportionately distributed due to disparities in access to care, differing environmental pollutant concentrations, temperature fluctuations, differing levels of stress, *etc.* Comparisons are possible between the Study Area, the city-wide average, Healthy People Objectives [20] and statewide comparisons. All comparisons must be made with the appropriate data qualifications including, for example, low counts resulting in statistically unstable hospitalization rates.

Socioeconomic indicators are also included and discussed in the Reference Document. Simple SES indicators such as percent of the population in a census tract below the poverty level and the percent of the population that is non-white may be compared to the county and statewide averages.

Several researchers [21–24] report associations between air pollutant concentrations and small for gestational age natality indicators. These small for gestational age indicators are included by zipcode and may be compared to county and statewide averages.

Access to healthcare is an especially important indicator of health outcomes. One index used to describe this vulnerability in a community is the percent of the population without health insurance. The data available for this indicator were not spatially refined. All of the communities within the city of Minneapolis were averaged, and various outlying suburbs were also reported. Comparisons for the Statute Area itself are limited, but the City of Minneapolis results can be compared to other geographic areas such as countywide averages [9].

The cardiovascular health indicator is rates of hospitalizations coded for ischemic heart disease. These data were included due to documented associations and presumed causality from increased levels of PM_2.5_. The spatial resolution of this data set is by zipcode, and comparisons to statewide data and countywide averages are possible.

### 2.8. Facility Specific Information

In the state of Minnesota, facilities with emissions of a single criteria pollutant over 250 tons per year are required to conduct an air emissions risk analysis [25]. An AERA may also be required at the discretion of the agency. Within the AERA process, facility emissions are modeled and resulting air concentrations are compared to health benchmarks. Summed non-cancer and cancer risk estimations from all emission units are compared to general risk assessment guidelines of 1 for hazard indices and 1 in 100,000 for cancer risks.

For the CL&E analysis, a screening multi-pathway risk analysis is conducted providing risk estimates with spatial coordinates [7,26]. The Statute Area is a densely populated urban area and thus the exposure scenarios considered are inhalation only, resident [12] and urban gardener. The urban gardener is a modified farmer [12] and includes inhalation, incidental soil ingestion, consumption of homegrown produce and consumption of homegrown eggs. This exposure scenario was developed by MPCA for incorporation into urban risk assessments due to growing interest in Minnesota for backyard egg production for personal consumption, and is used for the facility specific risk analysis required as part of the CL&E Analysis. The CL&E analysis provides the agency with the information needed to consider the potential facility-specific impacts, analyzed as described above, in a cumulative context with the existing conditions of the community. The CL&E data and analysis may be summarized as shown in Table 2.

### 2.9. Stakeholder Participation in the Method Development Process

During the development of the draft CL&E process, several small discussion groups were held with a variety of stakeholders, including those responsible for compiling the various data elements included in the Reference Document. These “technical check-ins” included academic faculty with expertise in relevant areas, community leaders, tribal technical staff, Hennepin County environmental health staff, Minnesota Department of Health staff, and a non-profit environmental group. The process was also presented to MPCA staff responsible for agency policy, small business assistance, air quality issues and those interested and active in making risk management decisions. The process was updated and greatly improved through the thoughtful input from these stakeholders. However, among community members there was an undercurrent of suspicion about working with government agencies.

Some community leaders voiced concerns that, should they contribute to this process (that might lead to a new permitted source in the community), there might be a perception that they had “sold out their community.” A large public informational meeting was held to present the first draft of the CL&E process and receive comments and suggestions from the community. Attendance was solicited by means of a targeted press release, emails to all neighborhood associations in the statute area, emails to the legislative authors, and flyers sent to community leaders who had attended the smaller technical check-ins. The planned meeting format followed suggestions by the community leaders and was organized into three parts: an open house with informational tables, a short 20 minute presentation with an extended question/suggestion/answer period, followed by a second opportunity to visit the informational tables and ask questions and provide comments and suggestions more informally. The themes of the informational tables included: the Statute Area definition and map, the statute language, the CL&E method and data used, air permitting within Minnesota, ambient air monitoring within the Statute Area, studies and information conducted and compiled by the MDH, and a sign-in table with a fact sheet available in English, Somali and Spanish. In addition to the opportunity to submit suggestions orally, a private suggestion box was included, as well as five large poster boards on the outside of the informational table area. Specific questions were written on these large poster boards including:

1. How can we notify you about future public meetings related to specific permit applications in this area? (e-mails, newspaper, posters/flyers, PCA website update, *etc.*)
2. What do you want to know about permit applications for proposed projects in this area (types of pollutants, estimated health risks, location of the project, who the company is, *etc.*)?
3. What community information should be included in a cumulative levels and effects analysis?
4. What concerns you about the environment or pollution in your neighborhood?
5. What was the most interesting, or surprising thing that you learned at this meeting?

Attendees were much more interested in the formal question/comment/answer period than perusing the informational tables. The question and answer period lasted longer than originally envisioned, and included complex and far-ranging questions. Some of these questions were: Are there other countries doing this?; Could we focus on avoiding hazards, rather than assessing risk?; Do environmental laws include background pollutant levels in the populations?; Who makes permitting decisions and how does that process work?; What is your educational background?; Does your agency require ethics courses?; and If MPCA can’t do the analysis described in law, then how can you issue any permit for this area?. The questions and comments reflected a desire to determine the credentials and trustworthiness of agency staff; the thoroughness of MPCA permitting procedures; a goal to stop all air permits within the area; a request for much more public input through additional informational meetings; a call to be shown in a map where “pollution deposits”; a desire for a cumulative risk level, body burden or an exposure level at which no more permits could be issued; a disbelief that cumulative risk assessment is complicated; a strong desire to have a voice in the permitting process; and an ability to review permit documents as they are received by the MCPA.

### 2.10. Limitations to the Methodology and the Public Participation Process

In addition to limitations noted elsewhere in the text, this CL&E methodology is further limited due to the scarcity of cumulative risk methodologies in general. Although some air toxics pollutants have multiple endpoints associated with their toxicity value, the scoping in this methodology (*i.e.*, presence and approximate radius of the Study Area) is based on facility-specific modeled air concentration results. There is a possibility, therefore, that a summed endpoint approach or other type of approach could result in a larger Study Area. Thus, although the final outcome includes consideration of cumulative environmental health data for all relevant health endpoints, the determined Study Area is the result of screening pollutants on an individual basis. Another limitation in this methodology is a lack of data directly connecting exposures in this community with the exact concerns voiced by the community members (e.g., neurodevelopmental effects from lead, asthma, heart disease, *etc*.).

## 3. Results and Implementation

Based on the input from the technical check-ins and the larger community informational meeting, the process was updated and the community outreach plan was adjusted.

### 3.1. Public Participation Plan for the Permitting Process

The MPCA developed a new approach for community outreach in response to questions and comments from community members, including a webpage, email notifications through GovDelivery^®^, permit application information sessions and public informational meetings.

The intent of the webpage is to support communication to interested stakeholders through posted documents and updates. The CL&E webpage includes the process and reference documents, a fact sheet (in Spanish, Somali and English), active permit applications, updates on active permit reviews and the opportunity to sign-up for GovDelivery^®^ notifications. The GovDelivery^®^ notifications provide updates of significant modifications made to the process or community outreach, updates on active permitting projects in the area, and dates and locations of informational sessions and/or public meetings.

Information sessions, similar to “office hours,” were incorporated into the public participation plan and are held when work on a permit application is started by the agency. In these sessions, the permit application materials and MPCA staff are available so that community members can review the permit application materials before a draft permit is developed. This is an opportunity for the community to ask questions about permitting and risk assessment in general as well as ask questions about the meanings of particular items in permit applications. The goal of these sessions is to provide community members with access to the permit application, and MPCA staff responsible for the review, to support community input during the application review process prior to the drafting of a proposed permit.

Finally, a public informational meeting is a mandatory part of the permitting process in the Statute Area. Generally, public informational meetings are only held if requested during the public comment period or if there is known controversy about a particular draft permit. The purpose of the public informational meeting is to present information concerning the draft permit, answer community questions about the draft permit, provide instructions for submitting comments on the draft permit and receive comments on the permit for those ready to do so.

### 3.2. Case Study of the First Permit Application

A permit application was received by the MPCA in early 2010, for a facility that includes a spray coating booth, natural gas combustion heating, maintenance activities, and an emergency generator. Under existing Minnesota rules, this type of facility would be eligible for a registration permit, which requires less agency review and is available to facilities with low actual emissions. However, because the facility was located in the Statute Area, the registration permit option was not available. Following the Process Document, the applicant conducted criteria pollutant modeling and air toxics modeling according to MPCA guidance. Screening levels were exceeded for the PM_2.5_ 24 hour SIL, the hourly NO_2_ SIL and the acute NO_2_ hazard quotient. The modeled one-hour average NO_2_ concentration exceeded the SIL at the furthest distance from the facility of all of the screening metrics. This distance defined the Study Area and extended to a radius of approximately 1.2 km from the facility fenceline.

The human health endpoint associated with short-term NO_2_ exposure is acute respiratory events, and therefore the following data were included, analyzed, and discussed in the CL&E report:

- traffic densities,
- acute respiratory air toxics modeling results,
- acute estimates for ambient air monitoring,
- asthma related hospitalizations,
- asthma related emergency room visits,
- results from the Asthma Capitals Study [27],
- smoking status rates [9],
- percent population uninsured [9],
- median income [28]
- percent of the population under the statewide average income [28], and
- the percent of the population that is non-white [28].

The human health endpoints identified for 24-hour PM_2.5_ exposure are acute respiratory events (described above) as well as cardiovascular health effects. The environmental health data used for an indicator of cardiovascular events were hospitalizations coded for ischemic heart disease.

The environmental health data for the Study Area were compared to data for the City of Minneapolis, the Twin Cities metropolitan area, Hennepin County and the State of Minnesota using the more refined spatial surrogate that the data would allow. Hospitalizations for asthma and cardiovascular events also included comparisons to Healthy People Objectives 2020 [29]. All modeled air concentrations were below facility-specific guidelines and state and federal standards. Multiple small natural gas-fired space heaters were the primary contributors to the modeled hourly NO_2_ concentrations. The spray booth and natural gas combustion were the primary contributors to PM_2.5_ emissions. These comparisons showing the relatively small impact of the proposed facility did not definitively answer the community’s main questions, which went beyond “Is this facility safe?” to the more difficult question, “Will this facility add to the preexisting environmental health issues that we already consider to be unacceptable?”. The facility-specific data and the existing community-based data were summarized, as demonstrated more generally, in Table 2.

Three information sessions were held in a local library during the technical review of the permit application, including a weekday evening, a Saturday morning, and a weekday noon. These information sessions were not well attended; however, those community members who did attend were able to speak directly with MPCA technical staff and the project proposers to clarify many details of facility operation and regulatory process. The project proposers also hosted a tour of the facility after one of the sessions. Community members at these sessions helped identify the need for “flag pole receptors,” or modeling receptors at locations of elevations higher than three stories due to the presence of multistory residential buildings in the Study Area.

Several factors were balanced during information sessions which occurred during the time when MPCA staff were conducting their technical review of the project. First, permit application materials were lengthy (hundreds of pages) and staff had not completed their review. Second, even for community members with technical backgrounds, some rule-based language is very specific in nature and can be difficult to decipher. Third, community members have “day jobs,” and they need to understand the information quickly in order to be able to participate and comment meaningfully. Fourth, community members expressed a mistrust of agency summaries of the permit application materials (complicated by the fact that the regulatory reviews were not complete). The difficult balance was between providing hundred page documents to the public in the very early stages, and summarizing information in a manner that would be both accepted and understood by community members. The informality of the setting and the availability of MPCA technical staff at the information sessions allowed the community to ask questions, obtain answers and make comments on the permit application. Thus, the sessions supported communication of technical information.

As regulatory review progressed, the MPCA made a determination that facility emissions under the proposed permit limits were unlikely to increase existing respiratory or cardiovascular health effects in susceptible populations. This determination was based on the risk assessment and CL&E analysis taking account of proposed facility operations, allowable air pollutant emissions, modeled concentrations, and all of the environmental health data that was reviewed during the process. The following questions were posed as a very basic risk management decision-making framework to determine whether to continue with the permit process:

1. Is the analysis adequate? (was all available data included, was this a reasonable “hard look” at potential facility impacts to existing cardiovascular and respiratory events, *etc.*)
2. Considering all of the information presented, would you recommend moving forward with a draft permit?
3. Are the limits incorporated into this analysis adequate to limit potential facility impacts to the community?
4. Are there any further voluntary efforts that the agency would suggest?

As part of the cumulative levels and effects analysis, the permit applicant proposed significant permit limits in order to reduce the size of the Study Area and the extent of the analysis. The resulting draft permit contained annual and daily coating usage limits in the spray booth, limits on contents of coatings used in the spray booth, annual limits on natural gas combustion and limits on VOCs and HAPs used in maintenance activities. During the permit application review, the facility applied for and received funding to incorporate geothermal heating which further reduces short-term emissions of respiratory irritants. This was not a requirement in the draft permit.

The required public comment period was extended from 30 to 45 days to allow greater community review of the draft permit. During the public comment period, a public informational meeting was held. A 20-minute presentation addressed the permit review process, the CL&E analysis, the requirements in the draft permit and the formal public comment process and was followed by a question and answer period. Questions were asked about existing versus future operation of the facility, odors, consideration of susceptible populations, why modeling is sometimes used instead of monitoring, why the facility was placed at this location, how compliance with permit limits is determined, the extent of current and future operational plans, and efforts to reduce energy consumption. Several commentors raised concerns about the environmental equity implications of siting the facility in a highly populated urban area, the desire for the site to be used for something other than a pollutant-emitting industry, that the meeting was not sufficiently publicized in alternative press outlets, and that the location of the public meeting was a deterrent for attendance because it was over a mile from the facility and outside of the Statute Area.

A second meeting was held to provide an additional opportunity for community review and comment. The majority of questions and comments at the second meeting were directed towards ensuring that the community has a voice in the process, the reluctance about having the facility in the neighborhood regardless of the CL&E results, the desire for community meetings with decision makers, and a disbelief in the information presented.

Formal written comments received during the comment period concerned the siting of the facility in a disadvantaged minority urban community rather than a suburb, requests for denial of the permit, a suggestion to assume synergy rather than additivity for pollutant interactions, requests that the MPCA Citizen’s Board make the permit decision, a desire to ensure that the existing condition was adequately considered, requests for additional time for community review, and requests for more community meetings.

Due in part to public comments, the proposed permit was presented to the MPCA Citizens’ Board for the final decision on issuance. The MPCA Citizens’ Board (Board) is appointed by the Minnesota governor, confirmed by the state senate, and chaired by the MPCA commissioner. Members of the public may submit additional written comments on the permit materials to the Board, may attend the meeting and may present at the meeting. Information on the general CL&E methodology was presented first, followed by the results of the facility-specific CL&E analysis and the proposed permit. The MPCA Citizens’ Board posed questions concerning the reasons for not pursing CL&E analyses at other locations and the resources needed to complete them. One board member noted that these analyses require a great deal of staff time to complete, but will get more efficient with practice. No community members attended the meeting, nor were additional requests or comments sent to the MPCA Citizen’s Board. In conclusion, the MPCA Citizen’s Board voted to authorize issuance of the permit.

## 4. Conclusions and Lessons Learned

The goal of the CL&E analysis is to examine the potential facility-specific impacts in the context of the existing condition of the community. The statute requirement is located within the Minnesota Statute authorizing the MPCA to issue air permits, therefore, the CL&E analysis is considered within the framework of permitting point sources of air pollution. Thus the CL&E analysis is focused on identifying the most likely facility contributions to potential cumulative impacts. Another way to think about the cumulative approach is that we are considering the totality of the existing situation into which a new action is proposed. The specific question in this case study was, given the existing stressors and vulnerabilities, will the addition of this new source be significant and should a permit be issued.

The regulatory framework for air permitting does not generally provide for consideration of factors such as existing conditions in the community surrounding a point source. This CL&E process requires review and consideration of data sources (e.g., socioeconomic indicators) not previously incorporated into permitting decisions. Further, results of a CL&E analysis may direct attention towards existing nearby facilities not currently under review or towards sources or stressors not under the direct regulatory authority of the agency (e.g., heavily trafficked roadways, *etc.*). Efforts should be made to incorporate environmental equity, as well as CL&E concepts, into analyses that inform air permitting; however, a comprehensive and systematic approach to managing air quality and risks is necessary. Communication between air permitting and more broadly focused programmatic staff is essential in incorporating CL&E findings into wider air pollution reduction strategies.

There was a perception among members of the community that the statute language would end permitting in the Statute Area thereby reducing air pollution in the community. This statute requires a more comprehensive permit review, but does create unique emissions limits for the area, set a threshold for estimated risks with respect to existing environmental health conditions, or establish a cumulative risk guideline. In the absence of specific rules limiting air emissions in high-ranking areas of potential environmental equity concern or disproportionately impacted areas, a CL&E analysis will provide additional information for making permitting decisions. The primary reason the facility proposed the permits limits was to limit the number of pollutants that screened in for further study as well as limit the size of the Study Area to be included in the CL&E analysis. Therefore, in this way some emissions reductions were gained through implementation of the statute.

In order to provide useful information and transparency, methods must be clearly written so that others (e.g., project proposers, consultants) can understand and apply them and community stakeholders can effectively participate. Data, both quantitative and qualitative, need to be publicly available and easily cited by third party contractors. In order to conduct cumulative risk analyses in areas with heightened environmental equity concerns, thorough “How To” documents must be written that include step-by-step directions and a public outreach component. The CL&E process required a comprehensive look combining facility specific analyses, environmental health data, and background data. Much of these data were qualitative and therefore difficult to convey clearly and transparently.

The ability of a governmental agency to share information depends in large measure on the success in building personal relationships between staff and community members. A lot of time, thought and practice was put into communication for the implementation of CL&E process and permit review, and still there was not likely enough time spent. By the end of the method development and later permit review cycle, the level of trust in the information and analysis being presented by MPCA staff was greatly improved, but trust in the decision-making function of the agency was still lagging. Communication of information is not possible in one single setting (such as one public meeting on a permit), especially information related to environmental equity and cumulative analyses. Only after multiple meetings and interactions with the same people were relationships built that made information sharing possible. Community members stated several times they did not care about the information being presented, they wanted to know if and how their voice made a difference in the decision making. Governmental agencies would be wise to consider policies and actions that foster relationship building with stakeholders before, during, and upon conclusion of permitting activities.

Each facet of the communication process, including seemingly simple things, required thought. Seat set-up was important for both the information sessions (people were more likely to congregate rather than sit down in front of a large permit document on their own) and the public informational meeting (MPCA staff needed to be on hand to answer questions but not in a way that would be perceived as an intimidating panel). Hand-held electronic devices are in constant and ubiquitous use. Community members must be reminded that all materials created by public employees are available upon request so that there is not a perception that information not immediately captured digitally may be lost. Furthermore, answers to questions may be immediately verified on these devices in real time. This gives credence to the long understood risk communication requirement of “tell them what you know and only what you know.” The option of several modes for providing input to the cumulative levels and effects process proved effective. Some community members prefer to write on poster boards, some prefer private notes and others prefer to speak publicly and be heard by all.

The scope and methodology of a cumulative risk analysis is dependent in part on population density. Two distinct cumulative risk assessment scenarios are apparent in general and represent the continuum from single source analyses to more complex considerations of cumulative context. The most simple is a rural to light suburban framework for new construction where one would consider the proposed facility and potentially ambient monitored background and/or the few individual nearby sources. This framework is the traditional large point source paradigm of considering human health risks in air pollution. However, in this case study within an urban environment other “nontraditional” sources were found to have a much greater impact on community health. In general, CL&E results may indicate that the majority of exposures are not from the facility in question nor may even within the typical point source regulatory paradigm. “Non-traditional” sources are sometimes a large number of very small emitters, and therefore do not fit well into the traditional large point source paradigm of human health risk assessment. Urban environments, therefore, are more appropriately addressed using CL&E type analyses with inclusion of multiple sources of data including hazard, exposure and health indices. However, acting upon the results to reduce potential human health risks requires broader policy implementation and much greater communication efforts.

## Figures and Tables

**Table 1:** Human health endpoints linked to environmental health data types for inclusion in Cumulative Levels and Effects Analyses.

	Acute (hourly exposure)	Chronic (lifetime exposure)
**Respiratory/Olfactory**	Traffic, Environmental Tobacco Smoke (ETS), criteria pollutants, Air Toxics, AQI, asthma data	Traffic, ETS, criteria pollutants, Air Toxics, AQI, asthma hospitalization data
**Developmental/Reproductive/Endocrine/Fetotoxicity**	Air Toxics, SMRSC site*	Air Toxics, drinking water, SMRSC site
**Hematological**	Air Toxics	Air Toxics
**Neurological**	Air Toxics	Air Toxics, mercury in fish, drinking water, SMRSE site, blood lead
**Eyes (irritant)**	Traffic, Air Toxics, AQI	Traffic, Air Toxics, AQI
**Alimentary**	Air Toxics, drinking water	Air Toxics, drinking water
**Bone & teeth**	Air Toxics	Air Toxics, drinking water, blood lead
**Cardiovascular**	Traffic, Air Toxics, AQI, ETS, criteria pollutants	Traffic, Air Toxics, AQI, SMRSE site, ETS, criteria pollutants
**Kidney**	Air Toxics	Air Toxics, drinking water
**Hepatic**	Air Toxics	Air Toxics, drinking water
**Cancer**	Not Applicable	ETS, traffic, criteria pollutants, Air Toxics, AQI, drinking water, SMRSC site, blood lead
**Ozone**	See respiratory endpoint above	See respiratory endpoint above
**Lead**		See neurological and carcinogenic endpoints above.
**Particulate Matter**	See respiratory endpoint above, and include cardiovascular data.	See respiratory endpoint above, and include cardiovascular data.
**CO**	See cardiovascular and neurological endpoints above.	Not Applicable
**NO_2_**	See respiratory endpoint above	See respiratory endpoint above, and include cardiovascular data.
**SO_2_**	See respiratory endpoint above	See respiratory endpoint above, and include cardiovascular data.

* Arsenic data from the South Minneapolis Residential Soil Contamination Site (SMRSC).

**Table 2:** General example for presentation of results for Cumulative Levels and Effects Reports.

	Specific Descriptors	General Discussion
**Existing Stressors**	• Ambient air toxics measurements	• Similar to other urban areas in St. Paul/Minneapolis
	• Ambient PM_2.5_ measurements	• Lower than National Standard, similar to other urban areas in St. Paul/Minneapolis
	• Traffic densities	• Similar to 10× statewide averages
	• Exposure to tobacco smoke	• Tied for highest smoking rates in metropolitan area
	• Potential exposures from nearby facilities (point sources)	• ~8 nearby facilities with potential exposures

**Descriptions of Vulnerabilities**	• Asthma hospitalizations and emergency room visits	• ~1.5–2 times higher than Minneapolis city-wide average
	• Cardiovascular hospitalizations	• High variability, uncertain
	• Socioeconomic status and minority populations	• Potential environmental equity area
	• Percent of Population without health insurance	• One of the higher in Hennepin County
	• Ranking in AAFA 100 Cities Asthma ranking	• Ranked best place in nation to live with asthma
	• Comparisons with Healthy People 2020 Objectives	• Asthma hospitalizations and ED visits in Study Area do not meet 2020 Healthy People objectives

**Pathways/media**	• Outdoor air, indoor air (ETS surrogate), ingestion of homegrown produce, incidental ingestion of soil	

**Routes**	• Inhalation, ingestion	

**Subpopulations**	• General population in the Study Area	• Consideration for children included (early lifestage exposure)

**Endpoints**	• Short-term respiratory and cardiovascular effects	

**Proposer Risk Reduction Activities**	• Geothermal heating	• Reduced NO_2_ emissions
	• Permit limits on daily and annual paint use	• Reduced particulate and VOC emissions
	• Permit limits on annual natural gas use	• Reduced NO_2_ emissions
	• Biofiltration gardens	• Reduced run-off from the site
	• Double panel filters on paint spray booth exhaust	• Reduced particulate emissions
	• Permit limits on specific metals in paints	• Reduced metallic emissions: chromium, lead, manganese, nickel or cadmium
	• Public transit is a lower impact activity than individual vehicles	• Reduced vehicle emissions (NO_2_, particulate)
